# An echocardiographic model for predicting the recurrence of paroxysmal atrial fibrillation after circumferential pulmonary vein ablation

**DOI:** 10.1002/clc.23712

**Published:** 2021-08-11

**Authors:** Yuxia Miao, Min Xu, Chunxu Zhang, Huannian Liu, Xiaoliang Shao, Yuetao Wang, Junhua Yang

**Affiliations:** ^1^ Department of Cardiovascular Division of The Third Affiliated Hospital of Soochow University Chang Zhou City China; ^2^ Department of Cardiovascular Division of The First Affiliated Hospital of Soochow University Su Zhou City China; ^3^ Department of Cardiovascular Division of Changzhou Municipal Hospital of Traditional Chinese Medicine Chang Zhou City China; ^4^ Department of Cardiovascular Division of Changzhou Cancer Hospital Affiliated to Soochow University Chang Zhou City China; ^5^ Department of Nuclear Medicine The Third Affiliated Hospital of Soochow University Chang Zhou City China

**Keywords:** catheter ablation, left atrium, paroxysmal atrial fibrillation, recurrence

## Abstract

**Background:**

Atrial fibrillation (AF) is a highly prevalent arrhythmia, with substantial associated morbidity and mortality. Circumferential pulmonary vein ablation (CPVA) is an effective rhythm control strategy, however, recurrence is an important factor influencing treatment decisions.

**Hypothesis:**

To develop a predictive model based on left atrial (LA) structure and function, and evaluate its efficiency in predicting the recurrence of AF after CPVA.

**Methods:**

Patients with paroxysmal AF who underwent CPVA were enrolled in this study and randomly divided into a development set and a validation set. The clinical and echocardiographic data of each patient were collected. In the development set, a least absolute shrinkage and selection operator (LASSO) regression was used to establish a LA ultrasound feature. By combining that LA ultrasound feature with independent clinical risk factors, we established an echocardiographic model using multivariate logistic regression and plotted the corresponding nomogram.

**Results:**

The LA ultrasound feature established by LASSO regression included nine echocardiographic indicators related to LA structure and function. It also exhibited good predictive ability in both the development set and the validation set (AUC:0.944, 95%CI: 0.910–0.978; AUC:0.878, 95%CI: 0.816–0.942). Logistic regression analysis indicated that LA ultrasound feature and AF duration were independent predictors for AF recurrence. The combined model including LA ultrasound feature and AF duration also showed good discriminability in both the development set (AUC: 0.950, 95% CI:0.914–0.985) and the validation set (AUC: 0.890, 95% CI: 0.831–0.949). The calibration curve showed good agreement between the predicted value and observed value.

**Conclusions:**

Our model that is based on LA structure and function measured by echocardiography is a useful non‐invasive preoperative tool, which exhibits good accuracy in predicting the recurrence of AF after CPVA.

## 1 INTRODUCTION

Atrial fibrillation (AF) is the most common clinical arrhythmia, with a population incidence of 1%–2%. There are about 30–100 million AF patients in the world.^1^ AF is also a progressive disease with an annual progression rate of 7%–15%,^2^ and more than 50% of paroxysmal atrial fibrillation (PAF) cases will develop into persistent atrial fibrillation (PeAF) within 10 years.^3^ Compared with PAF patients, the AF in patients with PeAF and more risk factors of cardiovascular disease usually progress faster to a more permanent form.^4^, ^5^ Therefore, positive rhythm control can slow down the progression of AF, as shown in a study where the progression rate of AF in the rhythm control set was 3.2 times lower than that in the ventricular rate control set.^5^ Circumferential pulmonary vein ablation (CPVA) is an effective rhythm control strategy. Multi‐center randomized clinical trials have found that CPVA works better than medications for treating PAF, providing a basis for using CPVA as a first‐line treatment for AF.^6^ The European Society of Cardiology 2020 recommends that AF catheter ablation for CPVA might be considered as a first‐line rhythm control therapy for improving symptoms in selected AF patients.^7^ However, the recurrence rate after CPVA is still high (up to 20%–40%).^8^ Therefore, it is important to establish a model to predict recurrence after CPVA in PAF patients.

Moreover, the study of risk factors based on the left atrial (LA) structure and function is an important part of the comprehensive management of AF. In AF patients, the original electrical and structural characteristics of the left atrium are remodeled, and this remodeling is the structural basis for recurrence after CPVA.^9^ Real‐time three‐dimensional echocardiography (RT3DE) is a new method for evaluating LA remodeling, and is more sensitive in determining LA dysfunction than other imaging modalities. The maximum LA volume (LAVmax) measured by 3‐dimensional (3DE) has independent and improved prognostic value over the LAVmax measured by 2‐dimensional echocardiography (2DE).^10^, ^11^, ^12^ The LA structure and function can be used to identify patients with a high risk of AF recurrence after CPVA.^13^, ^14^, ^15^ However, the LA indicators collected and calculated by echocardiography have multi‐dimensional collinearity, and the clinically significant factors may be screened out during the traditional logistics regression process. Therefore, the least absolute shrinkage and selection operator (LASSO) regression, a type of linear regression that uses shrinkage, was used to screen and establish algorithms based on LA indicators while achieving comprehensive variable selection and parameter estimation.

In our study, we used RT3DE technology to evaluate LA structural remodeling, and then performed a LASSO regression to obtain a LA ultrasound feature that represents various indicators of the left atrium. Furthermore, by combining the LA ultrasound feature with clinical risk factors, we established and verified a predictive model for AF recurrence after CPVA in PAF patients. Through this study, we hope to provide useful information for optimizing the rhythm control strategy of PAF patients.

## 2 MATERIALS AND METHODS

### 2.1 Objects

This is a single center, retrospective cohort study. The consecutive PAF patients who were hospitalized in The First People's Hospital of Changzhou City from October 2015 to December 2018 and underwent CPVA treatment for the first time were enrolled in our study. Inclusion criteria: (1) patients with AF confirmed by conventional electrocardiogram and/or 24‐hour dynamic electrocardiogram, and sinus rhythm could be restored spontaneously or after intervention within 7 days of onset; (2) at least one type I or III antiarrhythmic drug treatment was ineffective, or patient who was unable to tolerate the side effects of drugs and were willing to receive CPVA treatment. Exclusion criteria: (1) the image quality of 2DE was poor and cannot be further analyzed and processed; (2) AF shown by echocardiography; (3) left ventricular ejection fraction (LVEF) lower than 50%; (4) patients younger than 18 years old or older than 80 years old; (5) patients with structural heart disease; (6) transesophageal echocardiography (TEE) showed LA appendage (LAA) emptying velocity lower than 40 cm/s; (7) patients with acute or prior myocardial infarction; (8) patients with a history of thoracotomy; (9) patients with moderate to severe valvular dysfunction; (10) patients with chronic obstructive pulmonary disease and were treated with beta‐agonists; (11) patients with hemorrhagic constitution or intolerant to heparin and anticoagulant drugs. This study complied with the principles of the Declaration of Helsinki. All patients signed the written informed consent. This study has been approved by the Scientific Ethics Committee.

### 2.2 General information

By querying the medical record system, we obtained information on each patient including age, gender, body mass index (BMI), serum creatinine concentration (SCR), AF duration (the time from the first recorded electrocardiogram of AF to CPVA), smoking and drinking history, history of hypertension, diabetes mellitus (DM), congestive heart failure (CHF), stroke or transient ischemia attack (TIA), vascular disease (peripheral artery disease, or aortic plaque), hypertriglyceridemia (HTG), and other medical history or complications. The CHA_2_DS_2_‐Vasc score was calculated for each patient: CHF, hypertension, age ≥75 years (doubled), diabetes, stroke/transient ischemic attack/thromboembolism (doubled), vascular disease, age 65–75 years, sex category (female).

### 2.3 Image acquisition of echocardiography

Transthoracic echocardiography (TTE) and TEE were performed on the day before CPVA. All patients' images were collected during sinus rhythm. The inspection equipment was the Philips EPIQ 7C color Doppler ultrasound system (Philips Healthcare Royal Philips Electronics, Amsterdam, The Netherlands). All TTEs were performed using an X5‐1 probe by an attending physician who had more than 5 years of experience. The limb lead ECG was recorded while the patient was lying on their left side and breathing calmly. The routine M‐mode and 2‐DE images were measured. 2D images of 3–5 cardiac cycles at the parasternal left ventricular long axis, apical 4‐chamber, apical 2‐chamber, and apical left ventricular long axis were taken, with a frame rate >50 Hz. The end‐diastolic and end‐systolic endocardium of the left ventricle was traced on the 2D images of the apical 4‐chamber and apical 2‐chamber. LVEF was calculated using the biplane Simpson's method, and the average value was obtained from three measurements. The clear 2D image in the standard apical 4‐chamber view was acquired, and the “Full Volume” mode was used to place the LA endocardium fully in the sampling frame. After fixing the probe position and asking the patient to hold their breath, all dynamic images were stored on the hard disk in DICOM format for further offline analysis. To observe thrombus and spontaneous echocardiographic contrast (SEC) of the LA and LAA, all TEEs were performed using an X7‐2 probe by a senior certified echocardiography cardiologist. At the same time, the LAA peak emptying velocity was obtained from TEE.

### 2.4 Image analysis of echocardiography

Philips QLAB software (10.5, Philips Healthcare Royal Philips Electronics, Amsterdam, The Netherlands software pack) was used to analyze and obtain the 3‐dimensional image of the left atrium, as well as its volume and function parameters. On the end‐diastolic image of the apical 4‐chamber, the 3DQ‐A mode was selected, and the sampling point was placed on the LA wall. After adjusting the shape of the left atrium, we divided it into three parts, manually outlined the volume of each LA part, and calculated the minimum LA volume (LAVmin). Similarly, the end‐systolic image of the apical four‐chamber was used to calculate the LAVmax. The electrocardiographic image before the start of the P wave was used to calculate the LA pre‐systolic volume (LAVpreA). From these volumes, total, passive, and active emptying volumes and fractions were derived.^16^ The formulas for calculating the LA indices are as follows: expansion index (EI) = (LAVmax − LAVmin)/LAVmin, dilated emptying index (DEI) = (LAVmax − LAVmin)/LAVmax, active emptying percentage (AE) = (LAVmax − LAVpreA)/LAVmax, active emptying percentage index (AEI) = AE/body surface area (BSA), passive emptying percentage (PE) = (LAVpreA − LAVmin)/LApreA, passive emptying percentage index (PEI) = PE/BSA, LAVImax = LAVmax/BSA, LAVImin = LAVmin/BSA, LAVIpre = LAVpre/BSA. The BSA of each patient was calculated as follows: body surface area of male (m^2^) = 0.0057 × height (cm) + 0.0121 × body weight (kg) + 0.0882; body surface area of female (m^2^) = 0.073 × height (cm) + 0.0127 × body weight (kg) − 0.2106. All images were analyzed separately by two attending doctors, and the averaged values were used as final data. The two doctors were not informed about the study.

### 2.5 Circumferential pulmonary vein ablation procedure

Before the procedure, any antiarrhythmic drugs were stopped for five half‐lives and anticoagulation reached its standard level. The surgeries were performed under fentanyl analgesia. Coronary sinus electrodes were placed through the left femoral vein of the patient. Atrial septal puncture was performed via femoral vein under X‐ray fluoroscopy. Two Swartz sheaths were delivered into the left atrium. After selective pulmonary vein angiography, with the assistance of the Carto system (Biosense Webster, Diamond Bar, CA), a three‐dimensional anatomical model of the left atrium was established and circular linear bilateral pulmonary vein ablation was performed. After completion, the pulmonary vein segment with the most advanced pulmonary vein potential was searched under the guidance of the Lasso electrode, and the corresponding part on the ablation ring was supplemented for ablation. Radiofrequency ablation endpoint: complete disappearance of ipsilateral pulmonary vein potential or left atrium‐pulmonary vein conduction bidirectional block, and there is no recovery for at least 30 minutes. During the procedure, heparin was used to maintain the activated clotting time of whole blood at 250–300 seconds.

### 2.6 Postoperative treatment and follow‐up

All patients converted to sinus rhythm during surgery and completed their 3–6 months follow‐up. All patients took the previous anticoagulant for 2 months after the procedure, and then the medication was decided based on their stroke risk score. All patients continued to use an antiarrhythmic drug treatment for 3 months after surgery (amiodarone, 200 mg, Qd; propafenone 150 mg, Tid or morerazine 150 mg, Tid; sotalol 80 mg, Bid).Patients received a 12‐lead electrocardiographic examination in each week or when arrhythmia symptoms occurred, Moreover, patients returned to the outpatient clinic every month to record medical history, obtain physical examinations, and receive the 12‐lead electrocardiogram and 24‐hour dynamic electrocardiogram. All follow‐ups were conducted by personnel who had no information about their treatment plan. Any rapid atrial arrhythmia with ECG that occurred 3 months after CPVA surgery and lasted for more than 30s were defined as AF recurrence, which included AF, atrial flutter, or atrial premature contraction.

### 2.7 Statistical analysis

Normally distributed data were expressed as mean ± SD, and non‐normally distributed data were expressed as median and interquartile range. Comparisons between groups were performed using independent sample *t*‐test or Mann–Whitney *U* nonparametric test. Classification data were expressed as frequency or rate (%), and comparison between groups was performed by Pearson χ^2^ test or Fisher exact probability method. The LASSO regression was used for analysis, which is suitable for the regression of high‐dimensional data.^17^ By using the LASSO algorithm and 10‐fold cross‐validation parameter adjustment method, we selected the LA indicators with non‐zero AF recurrence coefficient after CPVA from the development set, weighted them by their respective LASSO coefficients, and obtained a linear combination of selected indicators. This formula was then used to calculate the risk score of AF recurrence after CPVA in each PAF patient (defined as LA ultrasound feature), which reflected the risk of AF recurrence. The predictive accuracy of the LA ultrasound feature was quantified by the area under the receiver–operator characteristic (ROC) curve (AUC) in both the development and validation sets.^18^

The logistic regression test was performed using AF recurrence as the dependent variable (recurrence = 1, no recurrence = 0), and the LA ultrasound feature and clinical variables of the development set as the independent variables. The likelihood ratio test with backward step‐down selection was applied to the multivariate logistic regression model. A nomogram was constructed based on the regression model. The AUC was calculated to quantify the discrimination ability of the nomogram. The calibration curve was plotted for both the development set and the validation set, and the consistency between observed and predicted values was compared.

All statistical tests were performed using R 3.4.1 (http://www.R-project.org/). The “glmnet” package was used for LASSO regression analysis. The “pROC” package was used to plot the ROC curve. The “rms” package was used for nomogram construction and calibration. All statistical tests were two‐sided, and *p* ≤ .05 was considered statistically significant.

## 3 RESULTS

### 3.1 The study flowchart

**FIGURE 1:**
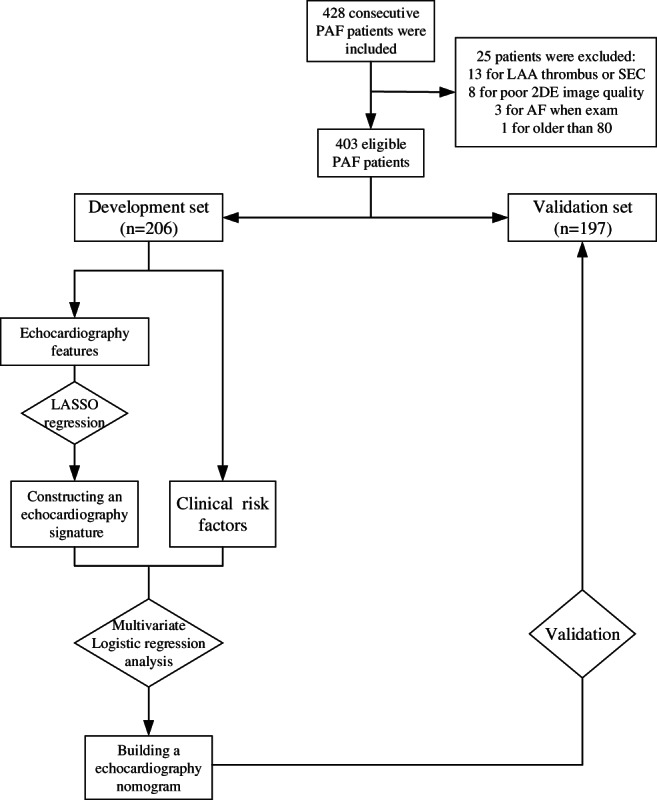
Flowchart of patients selection. 2DE, 2‐dimensional echocardiography; AF, atrial fibrillation; LAA, left atrial appendage; LASSO, least absolute shrinkage and selection operator; PAF, paroxysmal atrial fibrillation; SEC, spontaneous echocardiographic contrast

According to the inclusion criteria, 428 PAF patients were enrolled in this study. Among them, 13 cases were found to have LAA thrombus or SEC via TEE, eight cases had poor 2D images, three cases had AF rhythm, and one case was over 80 years old. Thus, these cases were excluded from the study. At last, 403 patients successfully underwent CPVA treatment, including 57 females and 346 males. The final study population was randomized on a 1:1 ratio into a development set (206 cases) and a validation set (197 cases) by the split‐sample function of the R software (Figure 1).

### 3.2 Comparison of general information between the development set and validation set

There was no statistical difference in general data between the two sets, including gender, age, LVEF, BMI, HR, SCR, AF duration, CHA_2_DS_2_‐VASc score, conditions of hypertension, DM, HTG, CHF, smoking history, drinking history, history of stroke, or TIA, vascular disease, and so forth. There was also no significant difference in LA structure and function indexes LAmax, LAmin, LApreA, EI, DEI, AE, AEI, PE, and PEI between the two sets (all *p* ≥ .05) (Table 1).

**TABLE 1:** Baseline characteristics of the development and verification sets

	Development set (*N* = 206)	Validation set (*N* = 197)	*p* value
Age (years)	63.03 ± 9.67	62.71 ± 9.64	.737
Male, *n* (%)	177 (85.92)	169 (85.79)	.969
LVEF (%)	55.57 ± 7.38	57.09 ± 22.46	.355
BMI (kg/m^2^)	22.40 ± 1.84	22.65 ± 2.07	.213
Heart rate (bpm)	77.63 ± 16.15	77.47 ± 15.76	.920
SCR (μmol/L)	60.95 ± 17.43	60.92 ± 17.82	.989
Hypertension, *n* (%)	58 (28.16)	52 (26.40)	.692
DM, *n* (%)	46 (22.33)	40 (20.31)	.620
CHF, *n* (%)	35 (16.99)	33 (16.75)	.949
Smoking history, *n* (%)	57 (27.67)	53 (26.90)	.863
Drinking history, *n* (%)	42 (20.39)	42 (21.32)	.818
HTG, *n* (%)	32 (15.53)	30 (15.23)	.932
History of stroke or TIA, *n* (%)	26 (12.62)	22 (11.17)	.652
Vascular disease, *n* (%)	18 (8.74)	20 (10.15)	.627
Antiarrhythmic drugs			
Amiodarone, *n* (%)	30 (14.56)	27 (13.71)	.805
Propafenone, *n* (%)	20 (9.71)	19 (9.64)	.866
Sotalol, *n* (%)	11 (5.34)	9 (4.57)	.722
β‐blockers, *n* (%)	94 (45.63)	87 (44.16)	.767
LAVImax (ml/m^2^)	32.38 ± 6.39	32.57 ± 5.40	.744
LAVImin (ml/m^2^)	17.68 ± 4.51	17.30 ± 3.39	.349
LAVIpreA (ml/m^2^)	24.97 ± 6.70	24.65 ± 6.45	.621
EI	0.86 ± 0.18	0.84 ± 0.14	.168
DEI	0.46 ± 0.06	0.45 ± 0.05	.099
PE	0.51 ± 0.19	0.53 ± 0.15	.276
PEI	0.24 ± 0.10	0.24 ± 0.06	.304
AE	0.49 ± 0.19	0.47 ± 0.15	.277
AEI	0.28 ± 0.08	0.28 ± 0.07	.360
AF duration (years)	7.90 ± 4.35	7.57 ± 4.08	.434
CHA_2_DS_2_‐Vasc	1.59 ± 1.19	1.55 ± 1.17	.740

Abbreviations: AE, active emptying percentage; AEI, active emptying percentage index; BMI, body mass index; CHF, congestive heart failure; DEI, dilated emptying index; DM, diabetes mellitus; EI, expansion index; HTG hypertriglyceridemia; LAVImax, maximum LA volume index; LAVImin, minimum LA volume index; LAVIpreA, pre‐systolic LA volume index; LVEF, left ventricular ejection fraction; PE, passive emptying percentage; PEI, passive emptying percentage index; SCR, serum creatinine concentration; TIA, stroke or transient ischemia attack.

### 3.3 Comparison between recurrence group and sinus rhythm group in the development set

In the development set, the patients were divided into a recurrence group (39 cases) and a sinus rhythm group (167 cases) according to the recurrence status. The LAVImax, LAVImin, LAVIpreA, and AE were higher in the recurrence group, and the EI, DEI, PE, and PEI were lower in the recurrence group. The differences were statistically significant (*p* < .001). The AF duration of the recurrence group was significantly longer than the sinus rhythm group (*p* = .003). The difference in other indicators was not statistically significant (Table 2).

**TABLE 2:** Comparison between recurrence and sinus rhythm groups in the development set

	AF recurrence (*N* = 167)	Sinus rhythm (*N* = 39)	*p* value
Age (years)	63.47 ± 9.27	61.15 ± 11.16	.178
Male, *n*(%)	142 (85.03)	35 (89.74)	.446
LVEF (%)	55.43 ± 7.49	56.16 ± 6.94	.574
BMI (kg/m^2^)	22.29 ± 1.85	22.92 ± 1.72	.053
Heart rate (bpm)	77.41 ± 16.22	78.56 ± 16.01	.688
SCR (μmol/L)	60.33 ± 18.62	63.59 ± 10.75	.295
Hypertension, *n*(%)	43 (25.75)	15 (38.46)	.112
DM, *n*(%)	38 (22.75)	8 (20.51)	.762
CHF, *n*(%)	27 (16.17)	8 (20.51)	.515
Smoking history, *n*(%)	50 (29.94)	7 (17.95)	.132
Drinking history, *n*(%)	36 (21.56)	6 (15.39)	.389
HTG, *n*(%)	25 (14.97)	7 (17.95)	.644
History of stroke or TIA, *n*(%)	21 (12.58)	5 (12.82)	.967
Vascular disease, *n*(%)	14 (8.38)	4 (10.26)	.709
Antiarrhythmic drugs			
Amiodarone, *n* (%)	26 (15.57)	4 (10.26)	.614
Propafenone, *n* (%)	16 (9.58)	4 (10.26)	.999
Sotalol, *n* (%)	7 (4.19)	4 (10.26)	.226
β‐blockers, *n* (%)	75 (50.30)	19 (48.72)	.723
LAVImax (ml/m^2^)	31.05 ± 5.70	38.04 ± 6.13	<.001**
LAVImin (ml/m^2^)	16.43 ± 3.48	23.02 ± 4.58	<.001**
LAVIPreA (ml/m^2^)	23.09 ± 5.36	33.050 ± 5.80	<.001**
EI	0.91 ± 0.15	0.67 ± 0.16	<.001**
DEI	0.47 ± 0.043	0.40 ± 0.06	<.001**
PE	0.55 ± 0.17	0.33 ± 0.15	<.001**
PEI	0.26 ± 0.08	0.13 ± 0.07	<.001**
AE	0.45 ± 0.17	0.67 ± 0.15	<.001**
AEI	0.28 ± 0.09	0.30 ± 0.07	.125
AF duration (years)	7.47 ± 4.00	9.72 ± 5.29	.003*
CHA_2_DS_2_‐Vasc	1.60 ± 1.15	1.56 ± 1.33	.870

* *p* ≤ .05;

** *p* ≤ .001;

Abbreviations: AE, active emptying percentage; AEI, active emptying percentage index; BMI, body mass index; CHF, congestive heart failure; DEI, dilated emptying index; DM, diabetes mellitus; EI, expansion index; HTG hypertriglyceridemia; LAVImax, maximum LA volume index; LAVImin, minimum LA volume index; LAVIpreA, pre‐systolic LA volume index; LVEF, left ventricular ejection fraction; PE, passive emptying percentage; PEI, passive emptying percentage index; SCR, serum creatinine concentration; TIA, stroke or transient ischemia attack.

### 3.4 Establishment of The LA ultrasound feature

**FIGURE 2:**
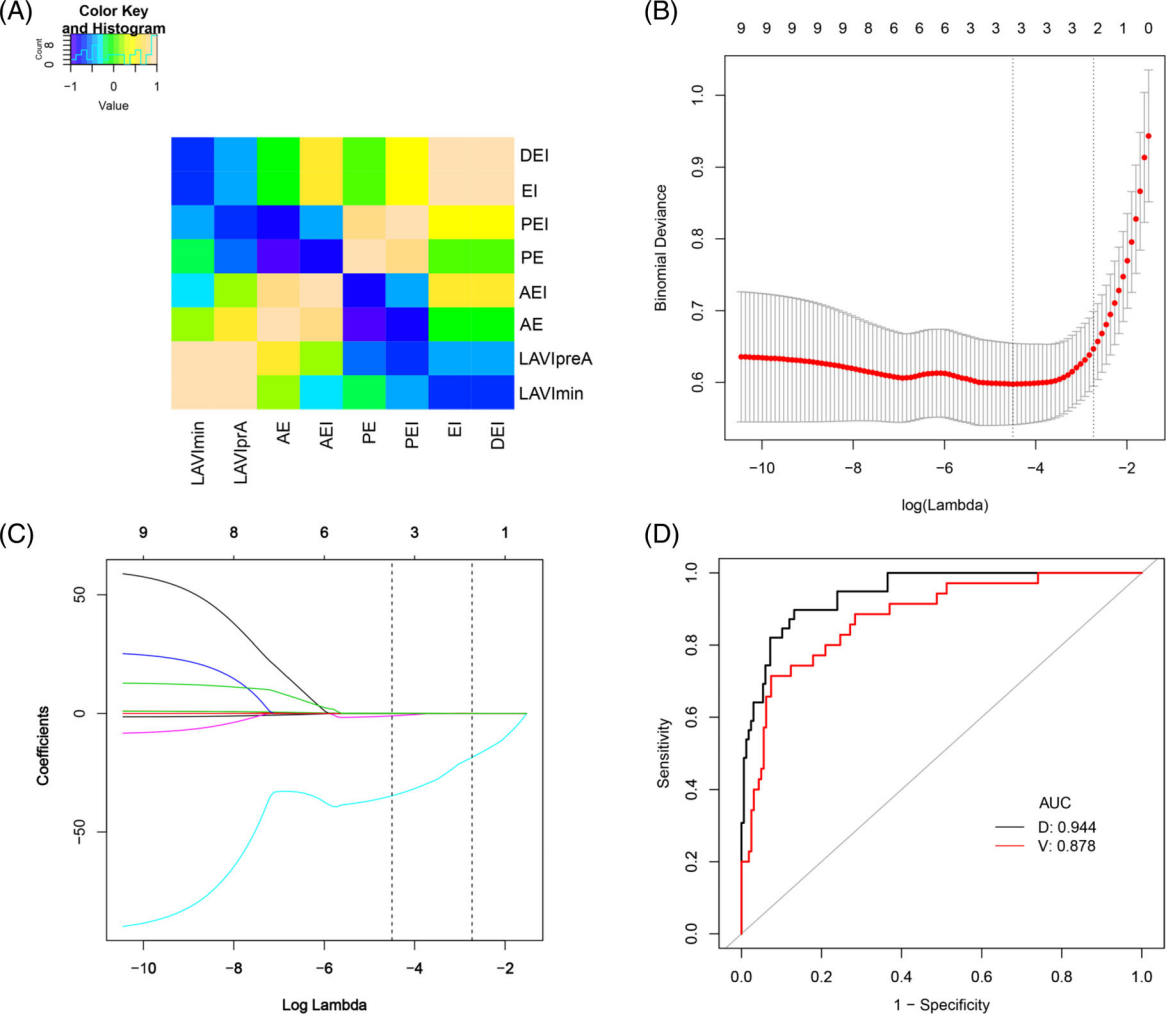
Selection of echocardiography features using LASSO logistic regression and its predictive accuracy. (A) There is multicollinearity among the variables of echocardiography. (B) Selection of the tuning parameter (λ) in the LASSO model via 10‐fold cross‐validation based on minimum criteria. Binomial deviances from the LASSO regression cross‐validation procedure were plotted as a function of log(λ). The y‐axis indicates binomial deviances. The lower x‐axis indicates the log(λ). Numbers along the upper *x*‐axis represent the average number of predictors. Red dots indicate average deviance values for each model with a given l, and vertical bars through the red dots show the upper and lower values of the deviances. The vertical black lines define the optimal values of l, where the model provides its best fit to the data. The optimal λ was selected. (C) LASSO coefficient profiles of the nine echocardiography features. The dotted vertical line indicates the value selected by 10‐fold cross‐validation in B. The nine features with nonzero coefficients are indicated in the plot. (D) The ROC curves of the LA ultrasound feature in the development and verification sets, respectively. AE, active emptying percentage; AEI, active emptying percentage index; D, development set; DEI, dilated emptying index; EI, expansion index; LAVImin, minimum LA volume index; LAVIpreA, pre‐systolic LA volume index; PE, passive emptying percentage; PEI, passive emptying percentage index; V, validation set

Nine LA size‐related features with nonzero coefficients were screened using a LASSO logistic regression model in the development set (Figure 2A–C). The LA ultrasound feature established by LASSO regression showed favorable predictive efficacy, with an AUC of 0.944 (95% confidence interval [CI]:0. 910–0.978) in the development set and 0.878 (95% CI:0.816–0.942) in the validation set (Figure 2D, Table S1). The difference of AUC between the two sets was not significant (*z* = 1.846, *p* = .065).

### 3.5 Comparison between the LA ultrasound feature and classic model using ROC curve

The predictive power of the LA ultrasound feature in the development set was better than the classic index LAVImax (AUC: 0.944 vs. 0.801), and the difference was significant (*z* = 3.656, *p* < .001). Moreover, the predictive power of the LA ultrasound feature in the validation set was also significantly better than LAVImax (AUC: 0.879 vs. 0.620, *z* = 3.727, *p* < .001) (Figure S1).

### 3.6 Model construction based on LA ultrasound feature established by LASSO regression and clinical features

**FIGURE 3:**
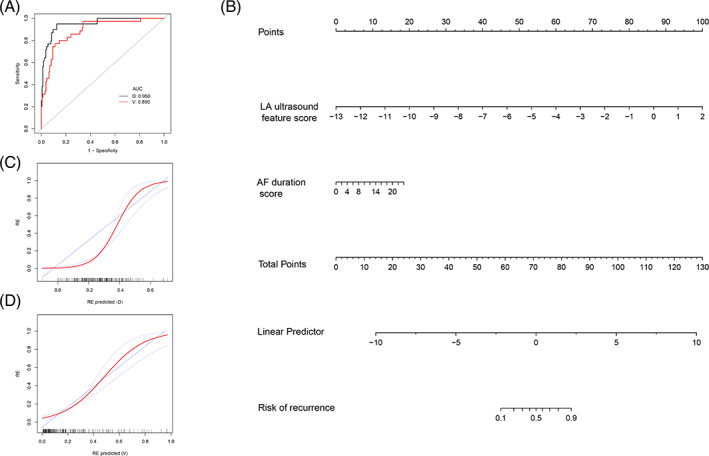
Plot (A) shows the ROC curves of the echocardiographic combined model in the development and validation sets, respectively. Echocardiography nomogram for the prediction of AF recurrence (B). Calibration curves of the echocardiography nomogram in the development set and validation set (C and D). Calibration curves depict the calibration of the nomogram in terms of the agreement between the predicted risk of AF recurrence and observed AF outcomes. The 45° line represents a perfect prediction, and the red lines represent the predictive performance of the nomogram. The closer the red line fit is to the ideal line, the better the predictive accuracy of the nomogram is. AF, atrial fibrillation; D, development set; LA, left atrial; V, validation set

The LA ultrasound feature and AF duration were identified as independent predictors of AF recurrence in PAF patients by a multivariate logistic regression model (Table S2). Therefore, we constructed a stepwise (stepAIC) selected model from the observed data: Logit(P) = 2.759 + 1.172* LA ultrasound feature+0.135*AF duration. The combined model also showed good discrimination ability in the development set (AUC: 0.950, 95% CI: 0.914–0.985) and validation set (AUC: 0.890, 95% CI: 0.831–0.949) (Figure 3A), but the predictive power was not significantly different from that using the LA ultrasound feature alone (*p* = .439; *p* = .384). An echocardiographic nomogram incorporating two predictors was then constructed (Figure 3B). The calibration curves of the development and validation sets show good agreement between the predicted value and observed value (Figure 3C,D).

## 4 DISCUSSION

Ablation is a well‐established treatment for symptomatic drug‐refractory paroxysmal AF, supported by a consistent body of evidence.^7^ However, radiofrequency energy can cause LA cell damage and necrosis, which can lead to LA scar tissue contraction. The postoperative AF recurrence not only accelerates the progress of AF, but also increases the economic and social burden of the patients. In this study, we developed a model that used the LA ultrasound feature to predict the probability of AF recurrence in PAF patients. With this model, we hope to find high‐risk PAF patients after ablation, improve the cost‐effectiveness ratio, and provide more precise clinical treatment plans for PAF patients. We performed LASSO regression to establish the LA ultrasound feature, which was applied to predict postoperative AF recurrence in PAF patients. The sensitivity and specificity of the model in the development set were 0.897 and 0.868, respectively, showing good discrimination ability.

Previous studies have found that the ampliative left atrium is a good predictor for AF recurrence.^19^ A meta‐analysis based on observational studies also indicated that the LAVi in patients with AF recurrence after ablation was significantly higher than the patients without recurrence.^20^ LAVImax measured by 2DE has become as an important biomarker for adverse cardiac events under a variety of cardiovascular conditions.^21^ In part because of the LA complex geometry and intricate fiber orientation, and the variable contributions of its appendage and pulmonary veins, quantifying LA size accurately was difficult previously. Recently, inaccuracies owing to geometric assumptions and foreshortening of the LA cavity with 2D biplane volume methods have been overcome with RT3DE, which has been shown to accurately and reproducibly estimate LAV compared with CMR.^22^ We demonstrated that the relapsed patients had higher LAVImax, LAVImin, and LAVIPreA measured by RT3DE than the non‐relapsed patients in the development set. We plotted the ROC curves of LAVImax and LA ultrasound feature in predicting AF recurrence after CPVA and compared them. By analyzing the ROC, we showed that the AUC of the LA ultrasound feature was significantly greater than LAVImax. Moreover, we used LASSO regression to analyze the data, which can process high‐dimensional data and retain the value of LA variables to the greatest extent. The diagnostic efficiency of LA ultrasound feature was found to exceed that of LAVImax.

In the early stages of AF, the atrium may increase its ejection capacity by increasing the preload (Frank–Starling mechanism). The first manifestation is an increase in volume, but its expansion capacity is limited. With frequent attacks, LA structure starts to remodel and leads to irreversible changes in LA function. The LA reservoir, conduit and booster pump functions can be calculated by both RT3DE and LA strain. The increased volume and dysfunction of the left atrium (decreased reservoir and conduit functions, and decreased or missing booster pump functions) are common in AF patients, which can be used to predict cardiovascular events.^23^, ^24^ Though the process of measuring strain by 2D speckle‐tracking echocardiography is relatively straightforward, methodological variations have contributed to the variation of reported normal ranges. Studies have shown that the LA storage function is independently related to the recurrence of AF after CVPA, which can further increase the risk classification of patients.^25^ Therefore, the LA storage function may reflect the degree of remodeling in PAF patients and serve as a predictor of recurrence at this stage. Our study showed that the LA storage function index DEI of the recurrence group was lower than the sinus rhythm group in the development set, suggesting that the structural remodeling induced by pressure or volume overload plays a very important role in AF recurrence.

The duration of AF was considered as an important prognostic factor for the outcome of cardiac rhythm after CPVA. The patients with a disease course of less than 2 years had a lower AF recurrence rate at 1 year after surgery than those with a disease course of more than 2 years,^26^ and the PeAF patients with a disease course of more than 3 years were more likely to relapse after CPVA than the patients with disease course of less than 3 years.^27^ We defined the time from the first recorded electrocardiogram of AF to CPVA as the duration of AF. By performing multivariate logistic regression, we found that, among the LA ultrasound feature that represented echocardiographic features and clinical risk factors, the AF duration was the only independent risk factor besides the LA ultrasound feature that affected the recurrence after CPVA in PAF patients. Moreover, according to the 2017 HRS/EHRA/ECAS expert consensus statement,^8^ we adopted a consistent rhythm monitoring program to monitor the recurrence of AF after CPVA.

The multivariate logistic regression model showed that AF duration and the LA ultrasound feature were independent risk factors for postoperative recurrence in PAF patients. The combined echocardiographic model that includes the LA ultrasound feature and AF duration showed good calibration and discrimination abilities (AUC: 0.950, 0.890) in both the development set and the validation set. However, compared with the LA ultrasound feature alone, the diagnostic efficiency of the combined model was not significantly different (*p* > .05). To provide clinicians with an easy‐to‐use tool, we constructed a nomogram based on a multivariate logistic regression model, which showed good calibration and recognition capabilities in both the development and validation sets. In practice, clinicians can calculate the total score using the scores corresponding to the variables of the LA ultrasound feature and AF duration in the nomogram, and draw a straight line down to the total score to obtain the corresponding recurrence probability. When categorized into low‐ and high‐risk groups based on the cutoff values of the risk score derived from the nomogram, the high‐risk group had a significantly greater probability of AF recurrence. Therefore, our nomogram can serve as an accurate and reliable predictive tool for AF recurrence after CPVA in PAF patients.

The limitations of this study mainly include the lack of external validation of the model, and the requirement for multi‐center validation with a larger sample size to obtain high‐level clinical application evidence. In addition, serological indicators were not included in our model. According to previous studies, the baseline BNP concentration in the serum of PAF patient might be a predictor of recurrence after successful electrical cardioversion.^28^

Our echocardiographic model is a non‐invasive predictive tool that combines the LA ultrasound feature and the AF duration. It showed good predictive accuracy of AF recurrence after CPVA in PAF patients. As a convenient and fast non‐invasive examination method, echocardiography can provide strong support to the comprehensive management of AF patients and the design of personalized treatment plans.

## CONFLICT OF INTEREST

All authors declare that: (1) they have not received any support from any organization having an interest in the submitted works, whether financial or otherwise; (2) there are no other relationships or activities that may affect the submitted works.

## Supporting information


**Data S1.** Supporting information.

## Data Availability

The data that support the findings of this study are available from the corresponding author upon reasonable request.
