# Supplementary material for: An echocardiographic model for predicting the recurrence of paroxysmal atrial fibrillation after circumferential pulmonary vein ablation
Source: Clin Cardiol. 2021 Aug 11;44(11):1506–15. doi: 10.1002/clc.23712 (PMC8571557; doi:10.1002/clc.23712)
Supplement: Supplementary file 1 — Data S1. Supporting information. [file CLC-44-1506-s001.docx]

Table_S1. Evaluation of the efficacy in predicting AF recurrence in development and verification sets using ROC curve

|  | AUC | 95%CI | Sensitivity | Specificity | PLR | NLR |
| --- | --- | --- | --- | --- | --- | --- |
| LA ultrasound feature(D) | 0.944 | 0.910–0.978 | 0.897 | 0.868 | 6.812 | 0.118 |
| LA ultrasound feature(V) | 0.878 | 0.816–0.942 | 0.714 | 0.926 | 9.643 | 0.309 |

Abbreviations: D, development set; V, validation set; LA, left atrial; AF, atrial fibrillation.

Table_S2. Multivariate analysis of risk factors affecting PAF recurrence

|  | Estimate | Std error | OR | 95%CI | *p* value |
| --- | --- | --- | --- | --- | --- |
| LA ultrasound feature | 1.172 | 0.194 | 3.227 | 2.207-4.718 | ＜0.001^**^ |
| AF duration | 0.135 | 0.065 | 1.145 | 1.009-1.299 | 0.036^*^ |

Note:***p*≤0.001 **p*≤0.05

Abbreviations: LA, left atrial; AF, atrial fibrillation; PAF, paroxysmal atrial fibrillation.


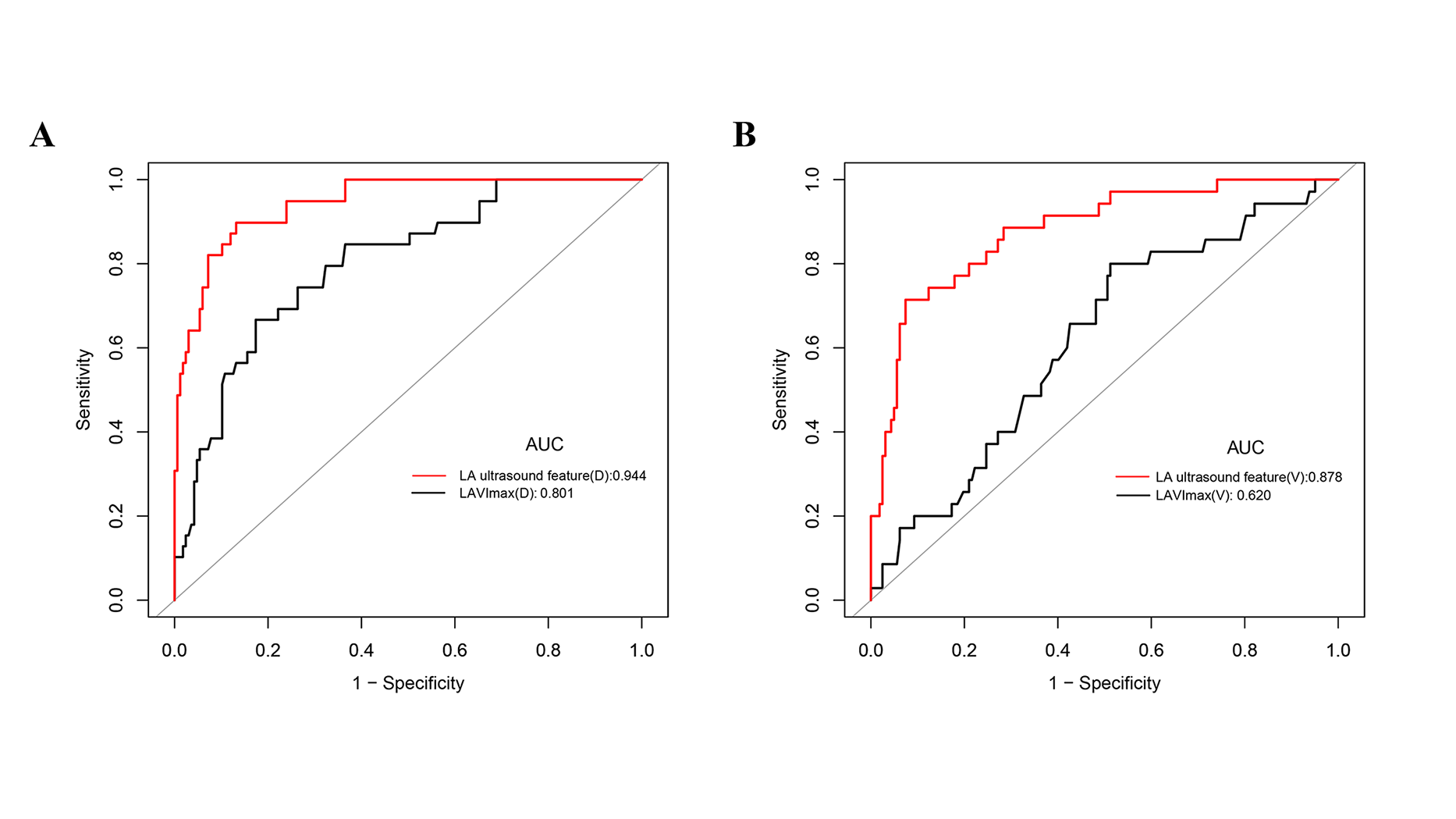


Figure_S1**.** Comparison between LA ultrasound feature and classic model using ROC curve. **A**: The ROC curves of the LA ultrasound feature and LAVImax in the development set. **B**: The ROC curves of the LA ultrasound feature and LAVImax in the validation set. D, development set; V, validation set; LA, left atrial; LAVImax, maximum LA volume index.
